# Legume Intercropping With the Bioenergy Crop *Sida hermaphrodita* on Marginal Soil

**DOI:** 10.3389/fpls.2018.00905

**Published:** 2018-07-02

**Authors:** Moritz Nabel, Silvia D. Schrey, Vicky M. Temperton, Lucy Harrison, Nicolai D. Jablonowski

**Affiliations:** ^1^Forschungszentrum Jülich GmbH, Institute of Bio- und Geosciences, IBG-2: Plant Sciences, Jülich, Germany; ^2^Institute of Ecology, Faculty of Sustainability, Leuphana University of Lüneburg, Lüneburg, Germany

**Keywords:** perennial energy crop, *Sida hermaphrodita*, marginal soil, legume intercropping, biomass production, bioenergy, facilitation

## Abstract

The cultivation of perennial biomass plants on marginal soils can serve as a sustainable alternative to conventional biomass production via annual cultures on fertile soils. *Sida hermaphrodita* is a promising species to be cultivated in an extensive cropping system on marginal soils in combination with organic fertilization using biogas digestates. In order to enrich this cropping system with nitrogen (N) and to increase overall soil fertility of the production system, we tested the potential of intercropping with leguminous species. In a 3-year outdoor mesocosm study, we intercropped established *S. hermaphrodita* plants with the perennial legume species *Trifolium pratense, T. repens, Melilotus albus*, and *Medicago sativa* individually to study their effects on plant biomass yields, soil N, and above ground biomass N. As a control for intercropping, we used a commercial grass mixture without N_2_-fixing species as well as a no-intercropping treatment. Results indicate that intercropping in all intercropping treatments increased the total biomass yield, however, grass species competed with *S. hermaphrodita* for N more strongly than legumes. Legumes enriched the cropping system with fixed atmospheric nitrogen (N_2_) and legume facilitation effects varied between the legume species. *T. pratense* increased the biomass yield of *S. hermaphrodita* and increased the total biomass yield per mesocosm by 300%. Further, the total above ground biomass of *S. hermaphrodita* and *T. pratense* contained seven times more N compared to the mono-cropped *S. hermaphrodita. T. repens* also contributed highly to N facilitation. We conclude that intercropping of legumes, especially *T. pratense* and *T. repens* can stimulate the yield of *S. hermaphrodita* on marginal soils for sustainable plant biomass production.

## Introduction

Cultivation of biomass crops on marginal soils requires careful design, establishment and maintenance of suitable cropping systems (Spiertz, 2013; Solinas et al., 2015). Marginal soils lack the nutrient resources necessary to provide a certain minimal productivity for plant biomass production and thus require a more costly input than what can be recovered by the output (European Environmental Agency, 2015). Physically, such soils may be sandy, rocky, shallow, low in plant-available nutrients and water and they need to be adequately prepared and maintained to support successful plant growth (Schröder et al., 2008). Thus, a suitable cropping system for particular local abiotic conditions needs to be implemented with regard to selecting the most appropriate plant species and cropping approaches.

Naturally occurring soils that are not suited for high-output agricultural production nevertheless harbor a distinct and well-adapted plant community of which perennial plants are an important part (e.g., grasslands on sandy soils; Loebel et al., 2015). Such plants often produce a deep- reaching root system, which enables access to water and nutrient reservoirs that are otherwise unreachable to roots of shallow-rooting plants. In addition, due to their long-lasting nature, roots of perennials store energy and N during dormant periods and then re-sprout early in the following growing season, allowing for high nutrient use-efficiency and recycling of N over time (Millard and Grelet, 2010; Voigt et al., 2012). Such N remobilization traits would be attractive options during the selection of plant species for biomass production on marginal soils.

A current prominent example of a perennial woody biomass plant is *Sida hermaphrodita* (Spooner et al., 1985); a species that has become a model plant for sustainable biomass production on marginal substrates in recent years especially in Poland (Borkowska et al., 2003; Barbosa et al., 2014; Nabel et al., 2016, 2017; Jablonowski et al., 2017). Following the establishment of the root system during the first growing season, *S. hermaphrodita* shoots emerge early in the next growing season from underground rhizomes which contain stored energy reserves (Borkowska and Molas, 2012). Early shoot emergence and the increasing amount of tillers with progressing cultivation time result in dense canopies reducing the necessity of weed and pest control and mechanical soil disturbance (Beare et al., 1994; Borkowska and Molas, 2012). Once established, the deep-reaching root system allows efficient exploitation of large volumes of soil for nutrients and water. Particularly on marginal soils, such root systems are beneficial for building soil structure (Bronick and Lal, 2005). However, in order to successfully cultivate *S. hermaphrodita* on a marginal substrate with the aim to produce ample biomass, adequate fertilization needs to be supplied to achieve the desired above-ground biomass production.

Compared to mineral fertilizers, organic fertilizers contain a high carbon concentration in addition to organically bound essential plant nutrients (Möller and Müller, 2012), which reduces the probability of nutrient leaching due to reduced mobility of the organically bound nutrients (Alburquerque et al., 2012a). Since marginal substrates are *per se* low in carbon content they would benefit from continuously applied organic fertilization which would gradually increase soil carbon content (West and Post, 2002). Digestate, a by-product of anaerobic fermentation of plant biomass for biogas production has been shown in our previous work to be a valuable organic fertilizer for *S. hermaphrodita* on marginal substrates (Barbosa et al., 2014; Nabel et al., 2014, 2017). The relative biomass increment of *S. hermaphrodita* fertilized with digestate continuously increased over three consecutive years compared to mineral fertilization, presumably due to the introduction of organic compounds and their additive effects on soil fertility (Nabel et al., 2017). Additionally, the re-introduction to the soil of nutrients that were removed with plant biomass during harvest and converted during biogas production, enables a cropping system that is independent of mineral fertilizers and allows closed nutrient loops, an essential concept of sustainable plant biomass production (Alburquerque et al., 2012b).

To stably provide N to such a cropping system, legume intercropping is commonly implemented in low-yield farming systems (Stagnari et al., 2017). The ability of legumes to fix atmospheric nitrogen (N_2_) is generally considered the reason for the experimental evidence that intercropping with legumes results in increased above-ground biomass production compared to mono crop cultures (Temperton et al., 2007; Roscher et al., 2012). With respect to biomass production on marginal soils, legume intercropping thus seems an obvious choice to create a more sustainable cropping system. Intercropping has further been described to produce earlier canopy closure, thus reducing the need for weed and pest control measures and also increases biodiversity, an important factor in every cropping system (Liebman and Dyck, 2009). Backing this up, in biodiversity ecosystem functioning experiments in grasslands, faster and more effective canopy closure occurs when more than one species is grown in a plot (Spehn et al., 2000). Most importantly, reduced need for mineral fertilizer renders the cropping system more economically viable since the main factor that determines the profitability is the cost for fertilization, in particular with N (Boehmel et al., 2008).

Interestingly, in our previous work we intercropped *S. hermaphrodita* with the highly efficient, deep-rooted N_2_-fixing legume *Medicago sativa* (with both species sown at the same time) and observed that this combination negatively affected *S. hermaphrodita* biomass compared to mono-cropping, even though total biomass of *S. hermaphrodita* combined with *M. sativa* was strongly stimulated (Nabel et al., 2016). Depending on the species combination, such an outcome is not uncommon since legumes, although bringing extra N into a system, are often strong competitors being fast growing (Malézieux et al., 2009). One of the mechanisms underlying improved biomass accumulation in intercropping is assumed to be complementary spatial distribution of roots within soil in more diverse mixtures resulting in differences in resource acquisition in either time or, more commonly, in space (von Felten and Schmid, 2008; Kahmen et al., 2012). Thus, using promising species combinations with complementary rooting depths (one shallow, one deeper- rooting species) could result in a vertical root orientation that circumvents direct competition for water and nutrients (Berendse, 1979). Thus, the careful selection of plant species regarding their root system traits and their composition with the aim of plant-specific complementarity could theoretically result in the identification of combinations of species with the potential to outyield compared to mono-cropping (Hoekstra et al., 2015; Hernandez and Picon-Cochard, 2016).

In the presented work, we aimed at an optimized *S. hermaphrodita* biomass production by intercropping with four different legume species (*M. sativa, Melilotus albus, Trifolium repens, T. pratense*), as well as a grass mixture as a control treatment. The aim of this study was to identify (i) if legume species selection and their respective N_2_-fixing abilities can result in outyielding the non-N_2_-fixing *S. hermaphrodita*, and (ii) to analyze whether this is reflected in total N content in the above-ground plant biomass and in the marginal substrate.

## Materials and Methods

### Study Site and Plant Cultivation

A 3-year outdoor mesocosm experiment was established at the Forschungszentrum Jülich GmbH (50°53′47′′ North and 6°25′32′′ East; 80 m a.s.l.) using 48 containers (40 cm × 40 cm × 40 cm), each filled with a sandy substrate (0/1 fine aggregate sand, RBS GmbH, Inden, Germany; TOC: 0 g kg^-1^; TN: 0 g kg^-1^; Ca: 0.3 g kg^-1^; K: 0.2 g kg^-1^; Mg: 0.8 g kg^-1^; P: 0.1 g kg^-1^), used as a model marginal substrate. The climate data during the experimental time from 2015 to 2017 are presented in **Table 1**. Besides the natural precipitation, mesocosms received additional irrigation via an automated drip irrigation system to prevent plants from severe drought stress (0.1 – 0.5 L day^-1^). Single seedlings of *Sida hermaphrodita* of BBCH stage 13–14 (Jablonowski et al., 2017) were transplanted into the mesocosms in April 2015. Legumes (alfalfa – *Medicago sativa;* white sweet clover – *Melilotus albus;* red clover – *Trifolium pratense;* and white clover – *Trifolium repens*; all purchased from Feldsaaten Freudenberger GmbH & Co. KG, Krefeld, Germany) as well as a commercially available grass mixture, not containing legumes (composed of 10% perennial ryegrass – *Lolium perenne;* 50% red fescue - *Festuca rubra;* 40% blue grass - *Poa pratensis; WB* 130 Mulchmischung III - Weinbergdauerbegrünung I; Feldsaaten Freudenberger GmbH & Co. KG, Krefeld, Germany) were sown (0.7 g mesocosm^-1^) in April 2016 into mesocosms containing the one year old *S. hermaphrodita* plants (*n* = 8). Additionally, eight mesocosms with *S. hermaphrodita* without intercropping were used as a control.

**Table 1:** Climate data for the outdoor mesocosm experiment: annual mean temperature and yearly precipitation values during the experimental time from 2015 to 2017 at the Forschungszentrum Jülich GmbH (50°53′47′′ North and 6°25′32′′ East; 80 m a.s.l.).

Year	Mean temperature (°C)	Precipitation (mm year^-1^)
2015	14.3	678.1
2016	14.9	666.3
2017	14.9	658.0

In May 2015, 2016, and 2017 all mesocosms received a fertilization using biogas digestate in a dose equal to a total N application of 160 kg ha^-1^. We chose this fertilization dose as it resulted in optimal plant growth in a previously published dose-response experiment for digestate fertilization of *S. hermaphrodita*, grown on the same marginal substrate used in this study (Nabel et al., 2014). The digestate was obtained from a commercially operating biogas plant using maize silage as feedstock (digestate dry matter mass fraction: 7.2%; N_total_: 0.53%; NH_4_^+^: 0.32%; P: 0.14%; K: 0.68%; Mg: 0.037%; Ca: 0.16%; S: 0.03%; organic matter: 5.3%, C:N ratio: 6; pH 8.2; all values refer to fresh weight; ADRW Naturpower GmbH & Co. KG, Titz-Ameln, Germany).

### Measurements

At the end of the growing season 2017, the aboveground biomass of *Sida hermaphrodita* and the intercropped species was harvested separately. Dry plant biomass was determined after drying at 70°C to constant weight. Additionally, soil samples were taken at 0–15 cm depth at the time of the biomass harvest and dried to constant weight at 30°C. Carbon (C) and nitrogen (N) content of the soil and the nitrogen concentration in the total aboveground plant biomass samples were determined by elemental analysis (VarioELcube, Elementar).

### Estimation of Effectiveness of Biological Nitrogen Fixation

In order to estimate the N_2_-fixation potential of the intercropped legume species on the marginal substrate, we invasively assessed nodulation of all legume species at the time of overall biomass harvest in October 2017 by extracting roots and assessing levels and quality of nodulation, following an ordinal scale-based field protocol of the British Columbia Ministry of Forestry, Canada (British Columbia Ministry of Forests, 1991). Soil cores of 20 cm depth and 7 cm diameter were taken to extract roots with nodules. The score took into account aboveground plant vigor (based on greenness of leaves and lack of wilting) and the number of nodules as well as nodule position, color, and appearance. The final score was then separated into three different possible categories that allow a swift assessment of nodulation efficiency: (1) effective nodulation (score: 20–25), (2) less effective nodulation (score: 15–20) or (3) not effective nodulation (score: 0–15), thus providing a rough indication of biological N_2_ fixation. This is a rough field method, but it allows to swiftly assess the effectiveness of nodulation (Nabel et al., 2016).

### Statistical Analysis

The experiment has a one factorial design with the intercropping factor separated into six levels: *Sida hermaphrodita* intercropped with one legume species each time (*Medicago sativa, Melilotus albus, Trifolium pratense*, or *T. repens*, respectively) or with grasses, as well as *S. hermaphrodita* grown alone as a control. Eight biological replicates were used for each treatment level. The collected soil samples were analyzed for C and N and plant samples were analyzed for N in four replicates. Statistical analysis was performed with analysis of variance (ANOVA) after trimming the data in R 3.0.3 (The R Foundation for Statistical Computing 2014) using the work package “Agricolae” (de Mendiburu, 2014).

## Results

### Biomass

**FIGURE 1:**
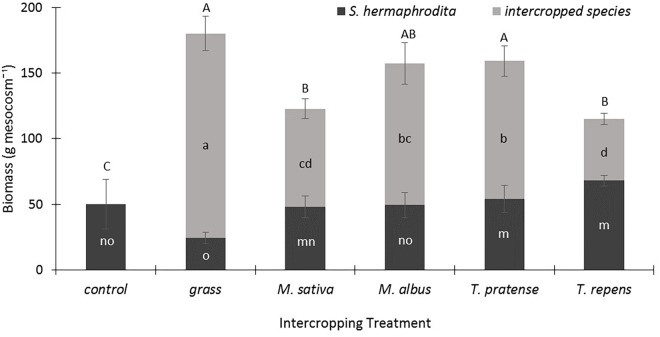
Total aboveground biomass is strongly increased by intercropping *S. hermaphrodita* with grass or legume species in outdoor mesocosms filled with sandy substrate. Control: Digestate fertilization of 40 t ha^-1^. Bars indicate the standard error (*n* = 8). Variants with the same letter are not significantly different (0.05 level). Capital letters indicate differences in significance of the total-above ground biomass. Small letters indicate differences in significance of the biomass of the individual species.

In their third year of growth, the *S. hermaphrodita* plants in the control treatment developed an average plant biomass of 50 g dry matter per mesocosm (**Figure 1**). Intercropping with the legumes *M. sativa* and *M. albus* did not result in changes of the *S. hermaphrodita* biomass, but increased significantly the total biomass yields per mesocosm by 100–200%. Intercropping of *S. hermaphrodita* with *T. pratense* and *T. repens* increased significantly the biomass yield of *S. hermaphrodita* by 8-15% compared with *S. hermaphrodita* mono-cropping, but *T. repens* also delivered the least additional biomass of all intercropped species. Grass produced the highest plant biomass dry matter yield of all intercropped species with 150 g mesocosm^-1^, while *S. hermaphrodita* biomass yield was significantly reduced compared with intercropping with *M. sativa, T. pratense*, and *T. repens* (**Figure 1**).

### Nodulation Assessment

All intercropped legumes had active nodulation with nodulation scores between 17 and 21.5 (**Table 2**). Both *T. pratense* and *T. repens* had scores in the range of “effective nodulation” while *M. sativa* and *M. albus* both showed scores in the range of “less effective nodulation” (British Columbia Ministry of Forests, 1991).

**Table 2:** Biomass and soil analysis of *Sida hermaphrodita* and intercropped species grown in outdoor mesocosms.

Intercropping Treatment	Intercropped species		*S. hermaphrodita*	Soil	
			
	Nodulation Score	Nitrogen (%)	Nitrogen (%)	Carbon (%)	Nitrogen (‰)
Dig (con)			1.0±0.1 a	0.8±0.1 b	0.7±0.0 a
grass mixture		1.1±0.0 c	0.4±0.0 b	2.1±0.1 ab	1.3±0.1 a
*M. sativa*	18.8±1.5 a	2.5±0.1 b	1.3±0.1 a	2.1±0.5 a	1.4±0.2 a
*M. albus*	17.3±1.0 a	2.3±0.1 b	1.2±0.1 a	1.4±0.2 ab	0.9±0.1 a
*T. pratense*	20.4±0.5 a	2.7±0.0 ab	1.3±0. a	1.7±0.4 ab	1.3±0.3 a
*T. repens*	21.5±0.4 a	3.2±0.1 a	1.2±0.1 a	1.2±0.1 ab	0.9±0.1 a

*The nodulation of legumes follows the “Field Guide to Nodulation and Nitrogen Fixation Assessment” of the British Columbia Ministry of Forests (1991). Score 0–14: no effective nodulation. Score 15–20: less effective nodulation. Score 20–25: effective nodulation. Dig (con): Digestate fertilization of 40 t ha^-1^without intercropping. n = 8 replicates for each treatment. ±Indicates the standard error. Different letters indicate statistically significant differences (p ≤0.05)*

### Nitrogen in the Plant Material

*Trifolium pratense* and *T. repens* showed N contents in their above ground biomass of 2.7 and 3.2%, respectively (**Table 2**). *M. sativa* and *M. albus* contained between 2.3 and 2.5% N while the grass mixture only contained 1.1% of N in the above ground biomass. The above ground biomass of *S. hermaphrodita* in the control treatment contained 1% of N. Intercropping with legumes increased N contents of *S. hermaphrodita* by 20–30%. Intercropping of *S. hermaphrodita* with the grass mixture resulted in 60% lower N content of the *S. hermaphrodita* above ground biomass.

**FIGURE 2:**
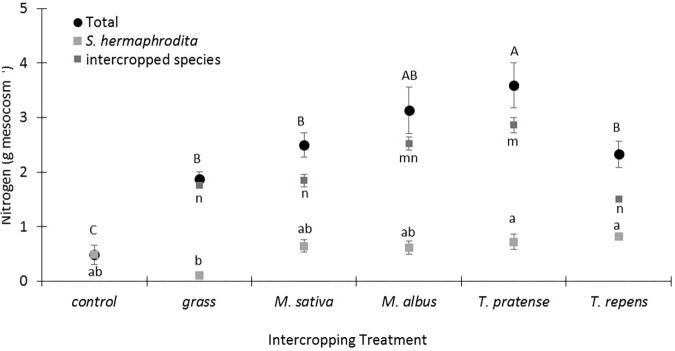
The total nitrogen content per mesocosm increased especially by intercropping *S. hermaphrodita* with *T. pratense*. Outdoor mesocosms filled with sandy substrate. Control: Digestate fertilization of 40 t ha^-1^. Bars indicate the standard error (*n* = 8). Variants with the same letter are not significantly different (0.05 level). Capital letters indicate differences in significance of the total nitrogen content per mesocosm. Small letters indicate differences in significance of the nitrogen content in the individual species.

Mesocosms of the control treatment without any intercropping contained the lowest content of total N in the above ground *S. hermaphrodita* biomass (0.4 g N mesocosm^-1^; **Figure 2**). Grass intercropping increased the total N in the above ground biomass fourfold compared to the control treatment, but this N was mainly found in the grass biomass. Treatments with legume intercropping showed the highest total N content in the above ground biomass per mesocosm with the highest value of 3.6 g N mesocosm^-1^ for *S. hermaphrodita* intercropped with *T. pratense*.

### Soil Analysis

After three growing periods, control-treated mesocosms, receiving digestate fertilization but no intercropping treatment, had a soil carbon concentration of 0.8% and a total N concentration of 0.7‰ (**Table 2**). No statistically significant difference in soil C or N between legume and grass intercropping treatments was found. The highest soil C (2.1%) and N (1.4‰) concentrations were found in mesocosms with *S. hermaphrodita* intercropped with *M. sativa*.

## Discussion

Intercropping of *S. hermaphrodita* with legumes generally increased the biomass yield per mesocosm, without reducing the yield of *S. hermaphrodita* compared to *S. hermaphrodita* mono-cropping, although *S. hermaphrodita* grown with the grasses showed the lowest aboveground biomass (**Figure 1**). In the concept of intercropping, a reduced yield of the individual species, but a higher total biomass yield would be expected (Malézieux et al., 2009). In our earlier study, we already tested the intercropping of *S. hermaphrodita* with *M. sativa* (Nabel et al., 2016). However, in this previous experiment we planted both species in the same year. This resulted in a clear advantage of *M. sativa* and a strongly reduced biomass of *S. hermaphrodita*. For the present study, we therefore created a priority effect by establishing *S. hermaphrodita* one year earlier than the intercropped species (see von Gillhaussen et al., 2014; Weidlich et al., 2017). This allowed *S. hermaphrodita* to be already more competitive against the intercropped species, promoting asymmetric competition in favor of the focus crop. Thus, the intercropped species did not negatively influence *S. hermaphrodita* yield compared to the mono-cropping of *S. hermaphrodita* (**Figure 1**). These findings agree with those of Weidlich et al. (2018) where maize grown with a legume neighbor grew as well as one maize plant growing without any neighbor, but where growth next to another grass species (wheat) reduced maize biomass significantly. Our results show no biomass reduction of *S. hermaphrodita* in the legume-intercropped treatments compared to mono-cropped *S. hermaphrodita* (**Figure 1**). This can possibly partly be explained by the fact that our experiments were performed in mesocosms, receiving fertilization and irrigation, reducing the yield-limiting factor of the resources water, light and nutrients. Due to the strong priority effect we found in our earlier study (Nabel et al., 2016), we consider the positive effect of adding the strongly N-facilitating intercrop after the establishment of the target crop *S. hermaphrodita* a promising approach, and indeed, it is a standard procedure in many intercropping systems.

Evidence for N facilitation usually includes either higher leaf N of intercropped species or higher aboveground biomass (Temperton et al., 2007). Technically speaking, since biomass is usually correlated with fitness and reproduction, one could argue that N facilitation has only occurred when biomass is stimulated. Overall, in our study intercropping with the four different legume species positively affected different traits of the *S. hermaphrodita* plants, but only the *Trifolium* species stimulated *S. hermaphrodita* biomass significantly (**Figure 1**). A finding that is also supported by the best nodulation status in the *Trifolium* species (**Table 2**). Seen through the strict facilitation lens, therefore, our study suggests using these two *Trifolium* species (i.e., *T. pratense* and *T. repens*) for *S. hermaphrodita* intercropping (Roscher et al., 2011). If one focuses on above ground biomass N, however, then the winning legume species for intercropping are *M. albus* and *T. pratense*. The species that most increases soil C and N on the other hand is *M. sativa*. This highlights the multifunctional roles different species play in a range of environments and the necessity to decide which ecosystem service one is most interested in fostering in a cropping system, e.g., biomass, carbon storage, shoot nutrient concentrations, or soil fertility. Overall though, it seems that *T. pratense* is an excellent all-rounder for intercropping with *S. hermaphrodita*, growing vigorously and fixing N at high rates. *T. repens* is also a good candidate since it shows high rates of N fixation with non-legume neighbors. However, its competitive ability is not as high as for *T. pratense* such that it would compete less with *S. hermaphrodita*. The higher yields for *S. hermaphrodita* growing with *T. repens* vs. *T. pratense*, not statistically significant though underline this finding.

During intercropping, the individual biomass of *S. hermaphrodita* increased when intercropped with either of the *Trifolium* species compared to the control treatment (**Figure 1**). These two legume species have a long history of successful use in extensive agriculture and are known to often transfer large amounts of their atmospherically fixed N_2_ to neighbors or subsequent crops, and have high potential for N_2_ fixation in mixtures (Roscher et al., 2011). Carlsson et al. (2009) found that *T. hybridum* and *T. repens* growing with grass neighbors in more diverse grassland communities increased the proportion of N derived from N_2_-fixation per biomass of plant compared to growing without grasses. This shows that competitive interactions can push the legume species to rely less on soil N and increase its N_2_-fixation reliance. Our study seems to find a similar effect, since the two legume *Trifolium* species that facilitated *S. hermaphrodita* during intercropping the most had the best nodulation scores.

In a similar experiment to our study, using the perennial energy crop switchgrass (*Panicum virgatum* L.) and the intercropping of different legume species including *T. pratense* and *T. repens*, Ashworth et al. (2015) also found a positive effect on biomass yield of the switchgrass. They explain this positive effect on yield by the legume-driven biological N_2_ fixation delivering additional N into the system.

The intercropped legume species all showed nodulation and *T. repens* and *T. pratense* were in the range of effective nodulation (British Columbia Ministry of Forests, 1991). These results are backed up by the results of the plant biomass N concentration, where *T. repens* and *T. pratense* both showed highest values of 3.2 and 2.7%, respectively (**Table 2**). In contrast, grass biomass only had a shoot N concentration of 1.1%. *T. repens* and *T. pratense* have been identified earlier for their high potential for N_2_ fixation in a 6-year grassland experiment, analyzing 12 different legume species (Roscher et al., 2011). Earlier studies indicated that neighboring species can also benefit from the biological N_2_ fixation of the intercropped legume and that one of the possible mechanisms is N sparing, whereby the reliance of the legume on N_2_ from the atmosphere leaves soil N (“spare N”) for the non-N_2_-fixing neighbors (Temperton et al., 2007). Especially for *Trifolium* species a high potential for this N-sharing and N-sparing was found (Frankow-Lindberg and Dahlin, 2013). Results of our study indicate the same, as *S. hermaphrodita* biomass showed a 20–30% increased N concentration when intercropped with the *Trifolium* species compared to the control treatment without legume intercropping. In contrast, grass intercropping reduced the N concentration of the above ground biomass by 60% compared to the control, indicating a strong competition for N (**Table 2**). Grasses like *Lolium perenne, Festuca rubra*, and *Poa pratensis* are known to be very strong competitors that effectively take up soil N (Ravenek et al., 2014).

The calculated total N in the above ground biomass illustrates these differences even more clearly as it combines the data of N content in the biomass of the individual species with the total biomass yield per mesocosm (**Figure 2**). Here we can show a clear difference between the total N content in the mono-cropped *S. hermaphrodita* control treatment and all intercropping treatments, no matter if a legume or grass was intercropped. We suggest that this effect can be explained by nitrate leaching out of the mesocosms, before *S. hermaphrodita* plants were able to take it up. Leaching of nitrate in the used model marginal substrate is a high risk as we could show in an earlier mesocosm experiment with *S. hermaphrodita* cultivated on sandy substrate (Nabel et al., 2016). Intercropping systems can be more effective in taking up N before it leaches out of the rhizosphere (Malézieux et al., 2009). Further, mesocosms in which *S. hermaphrodita* was intercropped with *T. pratense* contained more than 75% higher total N than mesocosms in which *S. hermaphrodita* was intercropped with grass. We relate this difference to the biological N_2_ fixation in *T. pratense*, while grass depends on the available N in the soil and rhizosphere. N derived from the atmosphere by biological N_2_ fixation can also be susceptible to leaching (Böhm et al., 2009; Warwick et al., 2016). Promising further steps in research would be to explicitly combine species in intercropping systems that have complementary root architecture (Malézieux et al., 2009). In the present study, the best performing intercropping species for *S. hermaphrodita* were *T. repens* and *T. pratense*, both of which have relatively shallow root systems, while *S. hermaphrodita* has a very deep reaching root system (Borkowska et al., 2009). In contrast, *M. sativa* and *M. albus* both have deeper reaching root systems like *S. hermaphrodita* and are therefore less suited for the intercropping with *S. hermaphrodita* since both may compete for the same local resources, especially in pots, limiting the maximum rooting depth. However, since plant biomass was analyzed after a total growth period of 3 years a reliable assessment of spatial root distribution was not feasible in the mesocosms we used with a maximum depth of 0.4 m. Therefore, field trials under agricultural conditions on marginal soils analyzing the dynamics of spatial root distribution of the used species combinations are now required to test whether our findings really were mainly driven by different root architectures and rooting depths.

An additional aspect of the more densely rooted soil is the potential for short term carbon storage in the soil (Steinbeiss et al., 2008a,b). We discussed the potential of an increased soil carbon content of marginal sandy substrate and the associated beneficial effects, like increased water holding capacity and soil respiration on soil fertility in an earlier publication (Nabel et al., 2017). Besides, the dense colonization of the top soil can reduce the high risk of erosion of the light sandy substrate (Liebman and Dyck, 2009).

## Summary and Conclusion

Intercropping of *S. hermaphrodita* with legume species can be an efficient way to increase the biomass output per unit area. We found no negative influence of legume intercropping on the biomass yield of *S. hermaphrodita* compared to mono-cropping. Legumes performed biological N_2_ fixation and thus enriched the cropping system with this essential nutrient. *S. hermaphrodita* benefited from the intercropping particularly when grown with *T. repens* or *T. pratense*. Highest biomass as well as highest total N content were reached when *S. hermaphrodita* was intercropped with *T. pratense*. Intercropping with *M. sativa* and *M. albus* still increased the total biomass yield but less effectively than intercropping with the *Trifolium* species. Further experiments could elucidate if these findings are mainly driven by differences in root architecture, with deeper rooting *M. sativa* and *M. albus*, being less complementary to the deep-reaching root system of *S. hermaphrodita*. When *S. hermaphrodita* was intercropped with grass, the latter caused strong competition for N. However, intercropping generally increased the N uptake, presumably reducing the risk of nitrate leaching in the light substrate.

The presented results were obtained from a model marginal substrate in mesocosms and are therefore not directly transferable into agricultural practice but indicate a promising possible direction for larger scale tests of such intercropping systems. Site-specific case studies in the field are now needed to test suitable combinations, such as those used in this study and others, to particular sites and management regimes (McWhinney, 2001). Results from such experiments could support the idea that efficient identification of growth traits of species can enable an optimal promotion of facilitation and niche complementarity resulting in better yields as well as more resilient systems (Hernandez and Picon-Cochard, 2016). We conclude that legume intercropping into perennial *S. hermaphrodita* energy crop cultures is an efficient way to increase the total biomass yield, and decrease the dependency on additional N fertilization whilst allowing organic C to enrich the soils, allowing for an extensive cultivation of marginal soils; specific legume species with not so deep rooting systems may be the best option for this when intercropping with *S. hermaphrodita*.

## Author Contributions

MN, SDS, VT, and NDJ designed the study. MN performed the main experiments and conducted the research under the supervision of SDS and NDJ. LH helped with experimental work and data acquisition. MN and SDS wrote the manuscript. All authors discussed the results, assisted in the manuscript preparation, and contributed to revisions.

## Conflict of Interest Statement

The authors declare that the research was conducted in the absence of any commercial or financial relationships that could be construed as a potential conflict of interest.
